# Opsoclonus Myoclonus Syndrome in a Case of Severe Acute Malnutrition in Children: A Case Report

**DOI:** 10.7759/cureus.32578

**Published:** 2022-12-15

**Authors:** Prachi Sharma, Shubhangi Patil Ganvir, Keta Vagha

**Affiliations:** 1 Department of Paediatrics, Jawaharlal Nehru Medical College, Acharya Vinoba Bhave Rural Hospital, Sawangi Meghe, Wardha, IND; 2 Department of Pediatrics, Jawaharlal Nehru Medical College, Acharya Vinoba Bhave Rural Hospital, Sawangi Meghe, Wardha, IND

**Keywords:** opsoclonus myoclonus syndrome, severe acute malnutrition, ataxia syndrome, myoclonus, opsoclonus

## Abstract

Opsoclonus myoclonus syndrome (OMS) is a rare (1 in 10 million people per year) but nonfatal autoimmune disorder characterized by involuntary oscillating eye movements, multifocal muscular jerky movements, severe ataxia, and neuropsychological and behavioral changes. It usually manifests as a paraneoplastic condition but has other etiologies also. To the best of our knowledge, this is the first case of OMS seen in a patient with severe acute malnutrition (SAM). Parents brought a three-year-old female child with complaints of being unable to sit, stand, and hold her neck for the past 18 months. The patient has had involuntary eyeball movements for three months and has shown regression in milestones. Our study aimed to understand the pathophysiology, etiology, and clinical course of OMS in a child with SAM. However, relapses and long-term developmental sequelae are common due to the lack of a common consensus regarding therapeutic guidelines.

## Introduction

Opsoclonus myoclonus syndrome (OMS) is a group of neurological signs and symptoms presenting simultaneously in an individual with rapid involuntary eye movements and jerky limb movements [1]. OMS is primarily an immune-mediated disease and is rare in presentation. It is multi-factorial in causation and presentation [1,2]. It is mostly idiopathic in etiology, followed by para-infectious and carcinomatous conditions. Neuroblastoma in children and breast carcinoma and small cell lung carcinoma in adults are the neoplastic conditions in which OMS presents as a paraneoplastic syndrome (PNS) [3].

It presents mostly within the first three years of life, making 18 months a median age of presentation. OMS has opsoclonus, myoclonus, and ataxia as its primary presentation [4]. Other presentations are irritability, mental retardation, and learning and behavioral disturbances in children in the long term. Attention deficits, visuomotor disturbances, severe cerebellar atrophy, and delayed developmental milestone achievement are less common but consistent clinical features.

Opsoclonus is defined as involuntary and oscillatory eye movements, which are omnidirectional and irregular oscillations [5]. They may range from a frequency between 6-15 Hz [6]. These eye movements also persist during sleep. Myoclonus is the irregular multifocal muscular jerks predominantly seen in trunk muscles. Ataxia is seen both in the trunk and limbs which presents as an inability to walk in an acute stage [5]. The rare presentation of OMS in a patient with severe acute malnutrition will throw light upon its multi-factorial cause and the need for a multidisciplinary approach both in terms of its investigations and management.

## Case presentation

A three-year-old female child, Hindu by religion, born out of non-consanguineous marriage, was admitted to our hospital with complaints of gradual regression of milestones noticed at 18 months of age and involuntary movement of eyeballs since three months. Parents complained of the child being unable to gain weight for five months. According to the mother, the child was in her usual state of health until she developed a low-grade fever that was not associated with chills and rigors and was relieved by taking medication. She had no history of vomiting and loose stools, cough, and cold. The child reported a history of weight loss with gradual regression of milestones, including, unable to hold her neck, sit or stand. There was a regression of language milestones; only disyllable words have been told by the child at present. Previously, the child's milestones were achieved according to the peer age group. The mother also noticed that the child had shown less interest in playing for three months, was lethargic, and did not respond well when played with. Antenatal, natal, and postnatal history were non-significant. Mother's age was 20 years when she conceived, and father's age was 22 years. The child was exclusively breastfed for six months, and weaning was done with biscuits, pulses, and mashed boiled vegetables, which have been continued till now. The mother noticed that the child also had involuntary movements of eyeballs like spontaneous, involuntary, arrhythmic, conjugate, and multidirectional saccades occurring in all directions of gaze. With the above complaints, the child was brought to the hospital for further management. On examination, the child was conscious and irritability was present. Her temperature was 99 degrees Fahrenheit; heart rate was 110 beats/min; respiratory rate was 24/min; oxygen saturation was 98%, and her random blood sugar was 76 mg/dl. There was no hypothermia or hypoglycemia. Anthropometry included height - 82 cm, weight - 7 kg, head circumference - 50 cm, and mid-upper arm circumference - 9.5 cm. According to the World Health Organization (WHO) classification, the weight for height was <-3 standard deviation, height for age was <-3 standard deviation, and this fell under marasmus. Prophylactically antibiotics were started. The appetite test was done in a comfortable place and was positive. On day 1, F-75 feed was started that is cow's milk 30 ml, sugar 9 gm (one level teaspoon), oil (1/2 teaspoon), and water to make 100 ml, so a total of 75 kcal in 100 ml, which was started at the rate of 130 ml/kg/day. Vitamin A was given 1 lac I.U., and as the child's weight was 7 kg, it was given on days 0, 1, and 14. Injection of magnesium sulfate 0.3 ml/kg, potassium 3 meq/kg/day, folic acid 1 mg/day, copper 0.2 mg/kg/day, zinc 2 mg/kg/day, iron 3 mg/kg/day was started. Routine investigations were done, the results of which are mentioned in a tabular form in Table 1 [7].

**Table 1:** Laboratory findings of the patient and the normal values. [7]

Investigations	Patient’s Values	Normal Values
Haemoglobin	11.7 g/dl	12-15 g/dL
Total Leucocyte Count	12500/mm³	4,000-11,000/mm³
Platelet	301,000/µL	150,000-450,000/µL
Hematocrit	33.50%	40-46%
Serum Urea	27 mg/dL	6-24 mg/dL
Serum Creatinine	1.3 mg/dL	0.74-1.35 mg/dL
Serum Sodium	131 mEq/L	135-145 mEq/L
Serum Potassium	3.7 mEq/L	3.5-5.5 mEq/L
Serum Calcium	8.3 mEq/L	8.5-10.2 mEq/L
Serum Magnesium	2.6 mEq/L	1.7-2.2 mEq/L
Serum Phosphorus	4.6 mEq/L	2.8-4.5 mEq/L

The liver function test and kidney function test were normal. Arterial blood gas was done and returned normal. Urine and stool routine microscope was done suggestive of normal. Creatinine kinase-33 and HIV card tests were negative. As the child was not taking oral food well, nasogastric tube feeding was started. The child was accepting feeding well, with no episodes of vomiting and edema and no history of convulsions. Significant weight gain was present, 10 g/kg/day. So F-75 feed has been changed to F-100 (cereal-based feed has been started). For OMS, the child was investigated for homovanillic acid (HVA), which was normal; a metaiodobenzylguanidine (MIBG) scan was done, which was normal, and a magnetic resonance imaging (MRI) of the brain was done, which was also normal. An electroencephalogram (EEG) was done, suggestive of normal. A cerebrospinal fluid (CSF) study was done where serum IgG and IgM were elevated. Antibodies like anti-neurofilament antibodies, anti-cerebellar antibodies, and anti-alpha-enolase antibodies have been found. The child was treated with corticosteroids like methylprednisolone with pulse therapy followed by a maintenance dose. It was started along with immunosuppressants cyclophosphamide. The eye movements were reduced as compared to her pre-intervention state, and the child tolerated feeds well with no refeeding syndrome. A significant weight gain of 256 g/day over five days of hospital admission was noticed, which summed up to an increase in weight of 1.10 kg as compared to that on admission. Tender loving care was given. The child improved gradually and hence was discharged.

## Discussion

The causative triggers for the development of OMS are still not clear. Most of the time, it is considered idiopathic in origin [2]. However, with the advancement of clinical knowledge, many theories are proposed forward which suggest different factors to be the etiology of OMS.

One such probable cause is parainfectious which primarily includes the infection by viruses. Mumps (paramyxovirus), coxsackie B3, rotavirus, and Epstein-Barr virus are some of the viruses which have been observed to be most commonly associated. Streptococcus, although less commonly seen to have a causal relationship, is also associated with the etiopathogenesis of OMS [8,9].

Another group of studies supports the paraneoplastic condition to be a contributor to its etiopathogenesis. Paraneoplastic OMS is almost exclusively associated with neuroblastoma in children and small cell lung carcinoma and breast carcinoma in adults [3]. Several studies have shown evidence of the role of autoantibodies in OMS. In children, which strongly suggests that the pathogenesis of OMS is autoimmune in origin [10]. Antibodies like anti-neurofilament antibodies, anti-Hu antibodies, anti-Purkinje cell cytoplasmic antibodies, anti-cerebellar granular cell surface antibodies, and anti-alpha-enolase antibodies are commonly found in the children of OMS. In adults, these are commonly seen in those who develop small-cell lung carcinoma or breast carcinoma, thus presenting as paraneoplastic syndromes. Some previous studies have found neurofilaments as a suspected autoantigen in OMS [11,12].

Inflammation in the brainstem and cerebrospinal fluid (CSF) involvement is seen in some children. A rise in the serum IgG and IgM levels and oligoclonal bands are seen. The B-cell activating factors have also been found to increase above their normal levels in the CSF of a pediatric patient suffering from OMS, which showed a decrease when treated with adrenocorticotropic hormone (ACTH) or steroids. No decrease was observed in children with OMS treated with intravenous immunoglobulin (IVIg) therapy [13,14]. Some researchers have proposed that brainstem dysfunction affects the omnipause neurons, which is a probable cause of OMS. Another group of clinicians suggests that a disbalance among the burst neurons and omnipause neurons in the brainstem and the fastigial neurons in the cerebellum is the pathogenesis in OMS [15,16]. Opsoclonus-myoclonus syndrome (OMS) is a group of neurological signs and symptoms presenting simultaneously in an individual [17]. It presents mainly within the first three years of life, making 18 months a median age of presentation. OMS has opsoclonus, myoclonus, and ataxia as their primary presentation. Other presentations are irritability, mental retardation, and learning and behavioral disturbances in children in the long term. Attention deficits, visuomotor disturbances, severe cerebellar atrophy, and delayed developmental milestone achievement are less common but consistent clinical features [4,5].

Opsoclonus is defined as involuntary and oscillatory eye movements, which are omnidirectional and irregular oscillations. They may range from a frequency between 6 and 15 Hz. These eye movements also persist during sleep [6]. Myoclonus is the irregular multifocal muscle jerks predominantly seen in trunk muscles. Ataxia is seen both in trunks and limbs and presents as an inability to walk in an acute stage. Most cases presenting with OMS were initially treated with corticosteroids (prednisolone, dexamethasone) or ACTH. The symptoms subsided partially in some patients but never completely recovered. Learning and behavioral impairments persisted. Withdrawal of these drugs caused the symptoms to relapse. Klein et al., in their study, have suggested that the IVIg regime in childhood OMS patients with relapses or steroid dependence be better in the outcome on the grounds of neurological signs [18]. Cyclophosphamide treatment in some patients has shown a ray of hope. When given along with ACTH and IVIg, monthly cyclophosphamide has shown better outcomes in stabilizing neurological manifestations. The relapse rate was lesser compared to individual usage of these drugs. In treating OMS, Rituximab, a monoclonal antiCD20 antibody, was first tried by Pranzatelli in 2005. It showed a substantial reduction in peripheral B-cells, and symptoms like irritable behavior and disturbed sleep also improved besides the motor functions.

Other treatment modalities like immunosuppressive drugs, plasma exchange therapy, and methotrexate are currently being used [19]. Supportive treatments like physiotherapy and neurological training are advocated to improve the myoclonus component [20].

## Conclusions

In this case report, a three-year-old female child presented with the chief complaints of inability to sit, stand, and hold her neck and regression of developmental milestones. The clinical picture pointed toward diagnosing opsoclonus myoclonus syndrome (OMS) with severe acute malnutrition (SAM). This case is unique because very few cases of such presentations have been reported. Through this case report, we intended to highlight that OMS can also be a feature of SAM. Currently, there is no well-accepted treatment for OMS, and intravenously administered methylprednisolone pulses and immunosuppressants can be used successfully in these patients for early recovery. Further scope of research is a requirement for treating and preventing relapses of OMS.
